# Adaptation and validation of the cancer awareness measure tool for individuals with intellectual disabilities in Hungary

**DOI:** 10.3389/fpubh.2026.1754719

**Published:** 2026-04-14

**Authors:** Mohammed Elmadani, Simon Klara, Godfrey Mbaabu Limungi, Maha Besbes, Amer Mesmar, Osama Hamad, Eva Horvath, Lívia Tóth, Orsolya Mate

**Affiliations:** 1 Doctoral School of Health Sciences, Faculty of Health Sciences, University of Pécs, Pécs, Hungary; 2 Department of Epidemiology, Faculty of Public Health, University of El Imam El Mahdi, Kosti, Sudan; 3 Jamhuriya Research Center, Jamhuriya University of Science and Technology, Mogadishu, Somalia; 4 Institute of Emergency Care, Pedagogy of Health and Nursing Sciences, Faculty of Health Sciences, University of Pécs, Pécs, Hungary

**Keywords:** cancer awareness, Hungary, intellectual disabilities, psychometric validation, screening participation

## Abstract

**Introduction:**

Cancer awareness is vital for early detection and prevention; however, individuals with intellectual disabilities (ID) often face challenges in accessing health information. The Cancer Awareness Measure (CAM) has not been tailored for this population. This study aimed to adapt and validate the CAM for individuals with ID in Hungary and to assess their cancer literacy.

**Methods:**

A cross-sectional study was conducted with 232 adults with mild and moderate ID living in community and residential settings. The CAM was simplified for cognitive accessibility, translated into Hungarian, and reviewed by the experts. The psychometric evaluation included internal consistency (Cronbach’s *α*), content validity (I-CVI and S-CVI/Ave), and face validity through pilot testing. The associations between cancer knowledge, barriers, and demographic variables were analyzed using independent t-tests and one-way ANOVA.

**Results:**

The adapted CAM showed satisfactory reliability across subscales (Warning Signs: *α* = 0.842; Risk Factors: *α* = 0.785; Prevention: *α* = 0.714; Barriers: *α* = 0.842) and excellent content validity (S-CVI/Ave = 0.96). Participants’ mean scores were 5.47/9 for Warning Signs, 36.41/55 for Risk Factors, 3.79/7 for Prevention, and 27.88/33 for Barriers to Seeking Help. Individuals living with family scored higher in warning sign knowledge (*p* = 0.022), while care home residents had higher barrier scores (*p* = 0.004). Those with a family history of cancer had higher warning sign (*p* = 0.002) and Prevention Knowledge (*p* = 0.047). Screening participation was associated with better warning sign knowledge (*p* = 0.02). Age ≥65 years, vocational education, and full-time employment were associated with higher knowledge scores. Sex differences were not observed.

**Conclusion:**

The Adapted CAM is a reliable and valid tool for assessing cancer awareness among individuals with ID in Hungary. The findings highlight demographic and contextual disparities, emphasizing the need for tailored, inclusive educational interventions and caregiver support.

## 1 Introduction

Cancer awareness plays a crucial role in the early detection and prevention of the disease, ultimately leading to reduced morbidity and mortality rates. Effective cancer prevention relies on a thorough understanding of the symptoms, risk factors, and importance of timely medical assistance for cancer. The CAM tool (1) has been developed globally to assess awareness levels across diverse populations, thereby identifying knowledge gaps and guiding targeted public-health interventions. Although the CAM tool is well-validated for the general population, no version has been specifically adapted or validated for individuals with intellectual disabilities (ID). ID is a neurodevelopmental condition marked by significant limitations in intellectual functioning and adaptive behavior, beginning before the age of 18 (2–8). Individuals with ID often face barriers to accessing healthcare information and services due to cognitive, communicative, and social challenges (8–11). Caregivers are pivotal in facilitating healthcare access for this population, supporting participation in screening programs, and aiding the understanding of health information (4). Despite similar cancer incidence rates between individuals with ID and the general population, this group experiences disparities in cancer outcomes, including lower screening rates, delayed diagnosis, and higher mortality rates for certain cancers (2–7, 10, 12).

Cancer awareness can also be understood within the broader framework of health literacy, which refers to the ability of individuals to access, understand, evaluate, and apply health information to make appropriate health decisions (13). Individuals with intellectual disabilities often experience lower levels of health literacy due not only to cognitive challenges but also to systemic factors such as inaccessible health communication, limited tailored educational resources, and exclusion from mainstream public health campaigns (8, 9). From the perspective of the social model of disability, these disparities are not solely the result of individual impairments but also arise from environmental and structural barriers that limit equitable participation in healthcare systems. Public health messaging, screening programs, and cancer education materials are frequently designed for the general population and may not be accessible for individuals with intellectual disabilities, thereby contributing to informational exclusion and delayed engagement with preventive services (8, 14). Situating cancer awareness within these broader frameworks highlights the importance of developing disability-inclusive assessment tools and health education strategies, such as the adapted CAM instrument evaluated in this study. It is important to recognize that individuals with intellectual disabilities represent a highly heterogeneous population, with substantial variation in communication abilities, educational experiences, living arrangements, and levels of support required for daily activities. These differences may influence access to health information, interactions with healthcare providers, and the ability to recognize and respond to cancer-related symptoms.

These disparities highlight the need for a dedicated and culturally adapted tool to assess cancer awareness among individuals with intellectual disabilities (ID). In the context of this study, cultural adaptation refers not only to linguistic translation of the instrument into Hungarian but also to ensuring that the questionnaire is accessible and meaningful within the lived experiences, communication abilities, and support environments of individuals with intellectual disabilities. Standard Cancer Awareness Measurement (CAM) tools are often inaccessible to those with ID due to their complex language, abstract concepts, or unsuitable response formats. Customizing these instruments allows for the reliable measurement of knowledge, attitudes, and perceived barriers within this population, offering unique insights into their educational needs and potential intervention strategies (8, 15–17). In Hungary, individuals with ID live in various residential and community settings, ranging from large institutions to small group homes and support housing initiatives (18–20). Large residential institutions often house many individuals with ID, sometimes within facilities originally designed for other populations, such as the Older population, reflecting historical classifications in the social care system (18). Smaller group homes and supported housing programs aim to promote independence and community integration but remain limited in their availability (19, 20). These diverse living arrangements, along with the variability in caregivers’ knowledge and support, underscore the need for a valid, accessible, and culturally tailored instrument to assess cancer awareness in this population.

Ensuring the psychometric validation of adapted instruments requires confirming their reliability, validity, and cultural relevance (16, 21–29). This process involves linguistic translation, cognitive accessibility, and item modification to match the population’s comprehension level (8, 16, 17, 23, 30). Previous studies in Hungary have successfully adapted various health-related questionnaires, such as the Peripheral Artery Disease Quality of Life (PADQOL) questionnaire and the STarT Back Screening Tool, highlighting the feasibility and importance of cross-cultural adaptation for accurate assessment (31, 32). Building on these methodologies, the adapted CAM tool for individuals with intellectual disabilities (ID) seeks to fill the gap in reliable cancer awareness assessments within this vulnerable group. This tool evaluates knowledge of cancer symptoms, risk factors, preventive measures, and barriers to seeking help. By providing a psychometrically validated measure, this study aimed to inform tailored health education programs, support caregivers, and guide public health policies to improve cancer outcomes for individuals with ID (6, 9, 33, 34). The primary goal of this study was to adapt and validate a CAM tool for individuals with intellectual disabilities in Hungary. The specific objectives included assessing the tool’s reliability within this population, evaluating the content and face validity of the adapted CAM tool, investigating cancer literacy among individuals with intellectual disabilities.

## 2 Materials and methods

### 2.1 Study design and participants

A cross-sectional study design was employed to adapt, simplify, and evaluate the internal consistency of the Cancer Awareness Measure (CAM) tool (1) for use among individuals with intellectual disabilities (ID) in Hungary. The primary objective of this study was to adapt and psychometrically validate the CAM tool for this population, providing a foundation for future large-scale investigations of cancer literacy among individuals with ID. The study included adults aged 18 years and older with mild or moderate intellectual disabilities residing in Hungary. This age group is particularly relevant due to their transition to legal healthcare autonomy, increased exposure to age-related health risks such as cancer, ongoing barriers to healthcare access, and the focus of many health promotion initiatives on adult populations (35–37).

Intellectual disability has traditionally been classified according to intelligence quotient (IQ) scores, typically categorized as mild (IQ 50–69), moderate (IQ 36–49), severe (IQ 20–35), and profound (IQ < 20) (38–43). In the present study, only individuals with mild to moderate intellectual disabilities were included, as participation required the ability to understand simplified survey questions and participate in structured interviews. Individuals with severe or profound intellectual disabilities were therefore not included due to the cognitive demands of the questionnaire.

Participants were recruited using a convenience sampling strategy through institutions and community organizations providing services for individuals with intellectual disabilities across Hungary. The Hungarian National Association of People with Intellectual Disabilities (ÉFOÉSZ) served as the primary recruitment partner. Recruitment was facilitated by local coordinators and caregivers who informed eligible individuals about the study and invited them to participate. Data collection sites included individuals with ID living with their families as well as residents from two residential care settings: ÉFOÉSZ Védőotthon in Andornaktálya, Heves County, and a supported family-style residential home located in the suburban area of Érd. ÉFOÉSZ Védőotthon provides mental health and social support services designed to promote residents’ independence and personal development. Residents have access to healthcare services, including general practitioners and psychiatric specialists, and receive assistance in obtaining both sheltered employment and opportunities in the open labor market.

### 2.2 Adaptation of the CAM tool

The CAM tool was originally developed and adapted in English between 2007 and 2008 by Cancer Research UK, University College London, King’s College London, and the University of Oxford to create a standardized method for assessing cancer awareness across diverse populations (1).

The original Cancer Awareness Measure (CAM) consists of 47 items, including recognition and open-ended recall questions assessing knowledge of cancer warning signs and risk factors. To improve accessibility for individuals with intellectual disabilities, several adaptations were implemented. First, open-ended recall questions were removed, as they require spontaneous recall and may increase cognitive burden for participants with intellectual disabilities. The adapted instrument therefore focused on recognition-based questions, allowing respondents to select answers from structured response options. After this modification, the adapted questionnaire contained 38 items.

Subsequently, the original CAM tool was simplified to improve accessibility for individuals with intellectual disabilities (ID). A team of experts, including public health specialists (*n* = 2), linguistic experts (*n* = 2), and tool specialists (*n* = 1), reviewed and adapted the tool using simplified language for clarity. Additionally, experts from the Hungarian Organization for Individuals with Intellectual Disabilities (*n* = 2) assessed the English version for cultural appropriateness. Based on feedback from experts, several modifications were made to the wording of the questions to enhance their clarity and ensure cultural relevance. The experts also suggested replacing open-ended questions with closed-ended questions and removing two questions related to knowledge of the most common cancers and the typical age of cancer onset. After these changes were implemented, a second review was conducted to ensure that the modifications were appropriately incorporated into the tool’s final version. The final adapted CAM tool consisted of four subscales and 38 items. The subscales included knowledge of warning signs of cancer (9 items, scored as Yes = 1, No = 0; maximum score = 9), knowledge of cancer risk factors [11 items, scored on a 5-point Likert scale (strongly agree = 5 → strongly disagree = 1; maximum score = 55)], knowledge of cancer prevention measures (7 items, scored as Yes = 1, No = 0; maximum score = 7), and barriers to seeking help related to cancer services (11 items, scored as Yes often = 3 → do not know = 0; maximum score = 33). Higher scores on the knowledge subscales (warning signs, risk factors, and prevention) indicate greater levels of cancer-related knowledge. In contrast, higher scores on the Barriers to Seeking Help subscale indicate greater perceived barriers to seeking medical care, reflecting more factors that may delay or discourage help-seeking behavior.

To enhance cognitive accessibility for individuals with intellectual disabilities, several linguistic and structural modifications were applied to the original Cancer Awareness Measure (CAM). Complex medical terminology and abstract phrasing were simplified to improve comprehension. For example, the original CAM item referring to *“persistent unexplained pain”* was adapted to *“pain that does not go away,”* and *“unexplained weight loss”* was simplified to *“losing weight without trying.”* Similarly, items describing potential warning signs were phrased using clear question formats such as *“Do you think a lump or swelling could be a sign of cancer?”* to facilitate understanding.

Response formats were also adjusted to improve usability. For the warning signs section, response options were simplified to Yes/No/Do not know, reducing cognitive complexity while maintaining the ability to assess knowledge of cancer symptoms. For the risk factor items, agreement scales were retained but presented with clear wording ranging from Strongly disagree to Strongly agree to support comprehension. Additional contextual questions were included to capture participants’ experiences with cancer screening, family history of cancer, and perceived barriers to seeking medical help. These adaptations were implemented to preserve the conceptual meaning of the original CAM while ensuring that questions were understandable and accessible for individuals with intellectual disabilities.

#### 2.2.1 Translation process

The original CAM tool was developed in the English language. Our process began by adapting and simplifying the English version, which was then translated into Hungarian by two native Hungarian translators fluent in both languages and with expertise in health communication and psychometrics. With the help of a linguist, the research team harmonized these translations into a single Hungarian version, addressing discrepancies. This procedure ensured that the final Hungarian version accurately reflected the meaning and cultural context of the original English version. Subsequently, the Hungarian translation was back-translated into English by two independent native Hungarian speakers fluent in English. The research team compared the back-translated version with the original English version to identify any discrepancies and made minor adjustments to clarify terms with different cultural connotations in Hungarian, where necessary. This back-translation and comparison process ensured the conceptual equivalence of the final Hungarian version of the scale. To enhance cognitive accessibility, the tool was further adapted by simplifying the vocabulary, reducing sentence complexity, and providing binary or simplified Likert scale responses. Cognitive interviews and focus groups with individuals with ID and their caregivers were conducted to improve understanding and ensure the acceptability of the tool (44, 45). The cognitive interviews, conducted 1 month before data collection, involved four participants and aimed to assess the clarity and comprehensibility of the questions asked. Concurrently, focus groups of seven participants were held to discuss the tool’s relevance, ease of use, and any challenges they encountered. Both the interviews and focus groups used semi-structured interviews and facilitator-led discussions to encourage open dialogue and capture a range of perspectives. Although feedback from these sessions provided valuable insights, no further refinements were made to the tool, as it was deemed sufficiently user-friendly and culturally appropriate for the ID population. Cognitive interviews were conducted with four individuals with intellectual disabilities to assess question clarity, comprehension, and response interpretation. Although the sample size was relatively small, cognitive interviewing studies focusing on questionnaire accessibility commonly employ small purposive samples because the primary objective is to evaluate item clarity and usability rather than statistical representativeness. In addition, a focus group involving seven participants provided feedback on the overall structure and comprehensibility of the questionnaire. Participants were able to interpret the simplified questions and response options without major difficulties. Feedback primarily related to the pacing of the interview process and clarification of instructions rather than the wording of specific items. Therefore, no substantial revisions to the questionnaire items were required following pilot testing.

#### 2.2.2 Expert committee review

To evaluate content validity, a diverse panel of experts, including linguists (*n* = 2), healthcare professionals (*n* = 3), ID specialists (*n* = 2), and individuals with ID (*n* = 4), reviewed all the versions of the tool to ensure semantic, conceptual, and cultural equivalence. The item content validity index (I-CVI) was calculated, retaining items with a score of ≥0.79 (46). This index was determined for all 38 items based on assessments by a panel of 11 experts who rated the items on a 4-point scale for relevance (1 = not relevant, 4 = highly relevant).

#### 2.2.3 Face validity

The Hungarian CAM underwent pilot testing with a group of 20 participants with ID to identify any ambiguities, comprehension difficulties, or logistical challenges. During this phase, semi-structured interviews were conducted with the participants to gather feedback on the clarity and usability of the tool. This feedback, along with observations from the testing process, enabled the evaluation of the tool’s suitability. Ultimately, no further refinements were deemed necessary as the tool was considered adequate for the target population (47).

### 2.3 Data collection and analysis

Data collection took place from October 2024 to September 2025 and was carried out by trained nursing staff and caregivers associated with the Hungarian Organization for Individuals with Intellectual Disabilities. To ensure standardization, all personnel underwent comprehensive training to adhere consistently to the research protocol, which included guidelines for conducting interviews and using accessible communication methods. Due to communication challenges, face-to-face interviews were preferred, with researchers using clear speech, allowing extra time, and providing reassurance to participants. Given the potentially sensitive nature of discussing cancer, interviews were conducted in a supportive environment using simplified language. Participants were informed that they could skip any question or stop the interview at any time. When appropriate, caregivers or staff members were present to provide reassurance or clarification. Standardized data collection materials, such as interview guides and Google Forms, were used at all sites to ensure uniformity. Data were collected using Google Forms; however, no personally identifiable information was recorded in the questionnaire. The dataset was therefore anonymized at the point of data collection. Access to the responses was restricted to the research team and stored on password-protected devices in accordance with institutional data protection regulations and ethical approval requirements. A total of 232 participants were recruited, and the sample size was deemed to be sufficient. To ensure consistency, data collection was closely monitored by site coordinators through regular check-ins to assess adherence to the protocol. The literature suggests that for calculating the Content Validity Index (CVI) and assessing the reliability and validity of the scale, a sample size of at least 200 participants is often sufficient to achieve reliable and generalizable results (48). Responses were transferred to Excel, cleaned, and imported into the SPSS (version 27) for analysis.

Prior to conducting parametric analyses, statistical assumptions were assessed. The normality of continuous variables was evaluated using the Shapiro–Wilk test and visual inspection of histograms, while Levene’s test was used to assess homogeneity of variances for group comparisons. Categories with extremely small sample sizes (e.g., civil partnership or “prefer not to say”) were excluded from inferential analyses or combined with similar categories to avoid unstable statistical estimates.

Content validation was assessed by computing the Item-Content Validity Index (I-CVI). Reliability analysis was conducted using internal consistency measured by Cronbach’s *α*. Descriptive statistics were used to explore the associations between participant characteristics and the CAM tool results. Independent sample *t*-tests and one-way ANOVA were used to analyze the associations between different variables and knowledge scores using means and standard deviations. Statistical significance was set at *p* ≤ 0.05.

## 3 Results

### 3.1 Internal consistency

The adapted Cancer Awareness Measure (CAM) demonstrated satisfactory internal consistency across its subscales (Table 1). The Warning Signs Knowledge subscale, consisting of nine items, showed good reliability (Cronbach’s *α* = 0.842), with a mean score of 5.47 (SD = 2.896); corrected item–total correlations ranged from 0.510 to 0.613, indicating strong internal consistency among the items. The Risk Factor Knowledge subscale, which included 11 items, demonstrated acceptable reliability (Cronbach’s *α* = 0.785) with a mean score of 36.41 (SD = 7.53), and corrected item–total correlations ranging from 0.292 to 0.610. The Prevention Knowledge subscale, comprising seven items, also showed acceptable internal consistency (Cronbach’s *α* = 0.714), with a mean score of 3.79 (SD = 2.05) and corrected item–total correlations ranging from 0.267 to 0.553. Similarly, the Barriers to Seeking Help subscale, consisting of 11 items, exhibited good reliability (Cronbach’s *α* = 0.842) with a mean score of 27.88 (SD = 9.38); corrected item–total correlations ranged from 0.286 to 0.642. Overall, these results indicate that the adapted CAM tool demonstrates adequate internal consistency and reliability for assessing cancer awareness and perceived barriers to seeking help.

**Table 1:** Internal consistency and item–total correlations of CAM subscales.

Subscale	Number of Items	Cronbach’s *α*	Corrected item–total correlation range
Warning Signs	9	0.842	0.510–0.613
Risk Factors	11	0.785	0.292–0.610
Prevention	7	0.714	0.267–0.553
Barriers	11	0.842	0.286–0.642

The dimensional structure of the instrument reflects the conceptual domains of cancer awareness. Items were grouped into four domains: warning signs, risk factors, prevention behaviors, and perceived barriers to help-seeking. Internal consistency analyses indicated that items within each domain demonstrated acceptable item–total correlations, supporting the multidimensional structure of the instrument.

Exploratory factor analysis using principal axis factoring with varimax rotation was conducted to examine the dimensional structure of the adapted questionnaire. The Kaiser–Meyer–Olkin measure of sampling adequacy ranged from 0.744 to 0.849 across subscales, indicating adequate sampling adequacy. Bartlett’s test of sphericity was significant for all domains (*p* < 0.001), confirming the suitability of the data for factor analysis.

The warning signs items loaded on two factors explaining 44.5% of the variance. The risk factor domain produced a three-factor structure explaining 40.4% of the variance. Prevention items demonstrated a single-factor structure accounting for 28.1% of the variance. Factor analysis of the barriers domain initially produced unstable estimates; therefore, a single-factor solution was retained consistent with the theoretical structure of the instrument.

### 3.2 Validity

The I-CVI values ranged from 0.79 to 1.00, with 25 items achieving an I-CVI of 1.00, indicating unanimous agreement on their relevance. Thirteen items scored between 0.79 and 0.99, which were deemed acceptable. No item was scored below 0.79. The average scale-level content validity index (S-CVI/Ave) was 0.96, indicating excellent overall content validity of the revised CAM tool.

### 3.3 Demographic characteristics

The study included 232 participants with an average age of 43.72 ± 12.61 years. A larger proportion of the participants were male (52.6%) than female (46.6%). Most participants were single or never married (70.7%), followed by those who were married or living with a partner (20.7%) and those who were divorced (4.3%). A significant number of participants had previous experience with cancer screening (57.8%), 27.6% had no previous experience, and 14.7% were unsure. Nearly half (48.3%) of the participants had a family history of cancer. Table 2 provides a detailed overview of the sociodemographic characteristics of the study participants (Table 3), which details the types of cancer screening conducted among individuals with ID, shows that gynecological (30.6%), lung (24.6%), and breast (17.9%) screenings were the most common, accounting for 73% of all reported screening activities.

**Table 2:** Sociodemographic characteristics of individuals with ID included in this study.

Variables		Frequency	Percent
Gender	Female	108	46.6
	Male	122	52.6
	Prefer not to say	2	0.9
Age	18–34 years	70	30.2
	35–49 years	82	35.3
	50–64 years	68	29.3
	65 + years	12	5.2
Marital status	Civil partnership	2	0.9
	Divorced	10	4.3
	Married/living with partner	48	20.7
	Prefer not to say	2	0.9
	Separated	2	0.9
	Single/never married	164	70.7
	Widow	4	1.7
Education	Below 8 grades	44	19
	8 grades	114	49.1
	High school	20	8.6
	Vocational school	38	16.4
	University/college	16	6.9
Place of living	Care home	158	68.1
	Family	74	31.9
Employment	Disability benefit	24	10.3
	Full-time job	44	19
	No job	17	7.3
	Part-time job	57	24.6
	Prefer not to say	82	35.3
	Retired	8	3.4
Cancer screening	Yes	134	57.8
	No	64	27.6
	Do not know	34	14.7
Family history of cancer	Yes	112	48.3
	No	120	51.7
^*^ Who would you turn to if you felt something unusual about your body?	Directly to the doctor	122	52.6
	To my helper/caregiver	124	53.4
	To my parents	44	19
	To my family member	42	18.1
	To my partner/friend	26	11.2

* Multiple answers were possible.

**Table 3:** Frequency and percentage of cancer screening programs reported by participants.

Screening type	Frequency	Percentage (% of 134)
Gynecological/cervical/cytology related	41	30.60%
Lung screening	33	24.60%
Mammography/breast	24	17.90%
Colonoscopy/colorectal/gastroscopy	13	9.70%
Prostate screening	7	5.20%
Mole/skin/dermatology	5	3.70%
Kidney/liver screening	2	1.50%
Other (unspecified mixed examinations)	9	6.70%
Total	134	100%

### 3.4 Association of CAM results and demographic characteristics

Table 4 highlights the differences in cancer knowledge and barriers between individuals residing in care facilities and those living with family members. Residents of care homes showed a significantly lower level of warning sign knowledge than those living with family (*t* = −2.303, *p* = 0.022), with a Cohen’s *d* of −0.324, indicating a small effect size. In contrast, no significant differences were found between the two groups regarding risk factor knowledge (*t* = 1.843, *p* = 0.067). However, individuals in care homes demonstrated significantly higher Prevention Knowledge (*t* = 2.121, *p* = 0.035) and greater Barriers to Seeking Help scores (*t* = 2.948; *p* = 0.004), with Cohen’s *d* values of 0.299 and 0.415, respectively, suggesting moderate effect sizes. Table 4 compares cancer knowledge and barriers between those who had undergone cancer screening and those who had not. Participants who had been screened for cancer exhibited significantly better warning sign knowledge (*t* = −2.338, *p* = 0.02), with a Cohen’s *d* of −0.301, indicating a small effect size. However, no significant differences were noted in risk factor knowledge (*t* = 0.076, *p* = 0.94), Prevention Knowledge (*t* = −1.672, *p* = 0.096), or Barriers to Seeking Help (*t* = −0.427, *p* = 0.67), with Cohen’s *d* values ranging from −0.057 to −0.222, indicating small to negligible effect sizes. Table 4 further examines individuals with and without a family history of cancer. Those with a family history of cancer possessed significantly higher warning sign knowledge (*t* = −3.09, *p* = 0.002), with a Cohen’s *d* of −0.406, indicating a moderate effect size. They also demonstrated significantly higher Prevention Knowledge (*t* = 1.996, *p* = 0.047), with a Cohen’s *d* of 0.261, suggesting a small effect size. Additionally, participants with a family history of cancer had lower Barriers to Seeking Help scores (*t* = 2.124, *p* = 0.035) than those without, with a Cohen’s *d* of 0.279, indicating a small effect size. Table 5 shows the gender differences in cancer knowledge and barriers to screening. Females had a higher mean score for warning sign knowledge (5.81) than males (5.23), though the difference was not statistically significant (*F* = 2.659, *p* = 0.072). No significant sex differences were identified for risk factor knowledge (*F* = 1.432, *p* = 0.241), Prevention Knowledge (*F* = 0.295, *p* = 0.745), or Barriers to Seeking Help (*F* = 1.193, *p* = 0.305), with Cohen’s *d* values ranging from −0.057 to 0.01, indicating negligible to small effects. Table 6 compares cancer knowledge and barriers across the age groups. Significant differences were observed for warning sign knowledge (*F* = 5.264, *p* = 0.002), with individuals aged 65 years and older scoring significantly higher (*M* = 8.00) than other age groups. However, no significant differences were found in risk factor knowledge (*F* = 1.76, *p* = 0.156), Prevention Knowledge (*F* = 1.14, *p* = 0.334), or Barriers to Seeking Help (*F* = 1.626, *p* = 0.184), suggesting a minimal age-related impact on these aspects.

**Table 4:** Comparison of Cancer Awareness Subscale Scores by living group, cancer screening participation, and family history of cancer (independent-samples *t*-test).

Sub-scale	Living group	*N*	Mean	SD	*t*	*p*-value	Cohen’s *d*
Warning Signs Knowledge	Care home	158	5.1772	2.7956	−2.303	0.022	−0.324
	Family	74	6.1081	3.0231			
Risk Factors Knowledge	Care home	158	37.0253	7.5864	1.843	0.067	0.26
	Family	74	35.0811	7.2806			
Prevention Knowledge	Care home	158	3.9873	2.0781	2.121	0.035	0.299
	Family	74	3.3784	1.9494			
Barriers to seeking help	Care home	158	29.1013	9.5004	2.948	0.004	0.415
	Family	74	25.2703	8.6092			
	Cancer screening						
Warning Signs Knowledge	No	98	4.9592	2.998	−2.338	0.02	−0.301
	Yes	134	5.8507	2.7707			
Risk Factors Knowledge	No	98	36.449	7.0229	0.076	0.94	0.01
	Yes	134	36.3731	7.9051			
Prevention Knowledge	No	98	3.5306	2.1692	−1.672	0.096	−0.222
	Yes	134	3.9851	1.9505			
Barriers to seeking help	No	98	27.5714	8.7107	−0.427	0.67	−0.057
	Yes	134	28.1045	9.8655			
	Family history						
Warning Signs Knowledge	No	120	4.9167	2.9179	−3.09	0.002	−0.406
	Yes	112	6.0714	2.7631			
Risk Factors Knowledge	No	120	37.25	7.8035	1.777	0.077	0.234
	Yes	112	35.5	7.1484			
Prevention Knowledge	No	120	4.05	2.188	1.996	0.047	0.261
	Yes	112	3.5179	1.8695			
Barriers to seeking help	No	120	29.1333	9.8808	2.124	0.035	0.279
	Yes	112	26.5357	8.6534			

*N*, sample size; SD, standard deviation, *t*, test statistic for the independent-samples *t*-test; Cohen’s *d*, effect size.

**Table 5:** Cancer Awareness Subscale Scores by gender (one-way ANOVA).

Outcome	Gender	*N*	Mean	SD	*F*	*p*-value
Warning Signs Knowledge	Male	122	5.23	2.77	2.659	0.072
	Female	108	5.81	3.00		
	Prefer not to say	2	2.00	0.00		
	Total	232	5.47	2.90		
Risk Factors Knowledge	Male	122	35.70	7.36	1.432	0.241
	Female	108	37.26	7.72		
	Prefer not to say	2	33.00	0.00		
	Total	232	36.41	7.53		
Prevention Knowledge	Male	122	3.87	2.04	0.295	0.745
	Female	108	3.72	2.10		
	Prefer not to say	2	3.00	0.00		
	Total	232	3.79	2.05		
Barriers to seeking help	Male	122	27.20	8.86	1.193	0.305
	Female	108	28.76	9.95		
	Prefer not to say	2	22.00	0.00		
	Total	232	27.88	9.38		

*N*, sample size; SD, standard deviation; *F*, variability between the groups.

**Table 6:** Cancer Awareness Subscale Scores by age groups (one-way ANOVA).

Outcome	Age	*N*	Mean	SD	*F*	*p*-value
Warning Signs Knowledge	18–34 years	70	5.14	2.75	5.264	0.002
	35–49 years	82	5.90	2.76		
	50–64 years	68	4.85	3.15		
	65 + years	12	8.00	1.04		
	Total	232	5.47	2.90		
Risk Factors Knowledge	18–34 years	70	37.46	7.58	1.76	0.156
	35–49 years	82	35.46	7.94		
	50–64 years	68	35.88	7.40		
	65 + years	12	39.67	2.23		
	Total	232	36.41	7.53		
Prevention Knowledge	18–34 years	70	3.66	2.22	1.14	0.334
	35–49 years	82	3.63	2.00		
	50–64 years	68	4.18	1.88		
	65 + years	12	3.50	2.32		
	Total	232	3.79	2.05		
Barriers to seeking help	18–34 years	70	29.06	9.68	1.626	0.184
	35–49 years	82	26.78	9.74		
	50–64 years	68	27.26	9.12		
	65 + years	12	32.00	3.95		
	Total	232	27.88	9.38		

*N*, sample size; SD, standard deviation; *F*, variability between the groups.

Table 7 presents the differences in cancer knowledge and perceived barriers according to marital status. Significant differences were observed across marital status groups for warning signs knowledge (*F* = 3.432, *p* = 0.018), risk factor knowledge (*F* = 5.263, *p* = 0.002), prevention knowledge (*F* = 3.905, *p* = 0.010), and barriers to seeking help (*F* = 5.343, *p* = 0.001). In general, married participants and those who were divorced or widowed demonstrated higher mean scores for warning signs knowledge compared with single participants. Conversely, single participants showed relatively higher scores for risk factor knowledge and prevention knowledge compared with other marital status groups. Differences were also observed in perceived barriers to seeking help, with single participants reporting higher barrier scores compared with married and divorced/widowed individuals. These findings suggest that marital status may be associated with variations in cancer awareness and help-seeking perceptions among the study participants.

**Table 7:** Cancer Awareness Subscale Scores by marital status (one-way ANOVA).

Outcome	Marital status	*N*	Mean	SD	*F*	*p*
Warning Signs Knowledge	Single	164	5.1	2.95	3.432	0.018
	Married	48	6.42	2.39		
	Divorced/widowed	14	6	3.46		
	Others	6	7	1.79		
	Total	232	5.47	2.9		
Risk Factors Knowledge	Single	164	37.15	7.23	5.263	0.002
	Married	48	34.71	7.75		
	Divorced/widowed	14	31	8.21		
	Others	6	42.33	2.07		
	Total	232	36.41	7.53		
Prevention Knowledge	Single	164	4.05	1.99	3.905	0.01
	Married	48	3.17	2.22		
	Divorced/Widowed	14	2.71	1.33		
	Others	6	4.33	2.25		
	Total	232	3.79	2.05		
Barriers to Seeking Help	Single	164	28.87	9.06	5.343	0.001
	Married	48	25.54	9.64		
	Divorced/widowed	14	21.29	9.13		
	Others	6	35	5.59		
	Total	232	27.88	9.38		

*N*, sample size; SD, standard deviation; *F*, variability between the groups.

Table 8 details the results for cancer knowledge and barriers based on participants’ educational attainment. Significant differences were found in warning sign knowledge (*F* = 4.194, *p* = 0.003), with those who completed vocational school or higher education showing better knowledge. For Prevention Knowledge, significant differences were noted (*F* = 2.691, *p* = 0.032), with individuals with less than eight grades achieving higher average scores. However, no significant differences were detected in risk factor knowledge (*F* = 2.023, *p* = 0.092) or Barriers to Seeking Help (*F* = 2.064, *p* = 0.086). Table 9 highlights the variations in cancer knowledge and barriers across various employment statuses. Significant differences were observed in warning sign knowledge (*F* = 7.944, *p* = 0.000), with full-time employees achieving notably higher scores than those in other employment categories. Similarly, risk factor knowledge (*F* = 11.331, *p* = 0.000) and Prevention Knowledge (*F* = 4.265, *p* = 0.001) were significantly higher among full-time workers. Significant differences were identified in Barriers to Seeking Help (*F* = 14.399, *p* = 0.000), with the part-time workers reporting the lowest scores.

**Table 8:** Cancer Awareness Subscale Scores by educational level (one-way ANOVA).

Outcome	Education	*N*	Mean	SD	*F*	*p*
Warning Signs Knowledge	Below 8 grades	44	5.68	2.87	4.194	0.003
	8 grades	114	4.75	2.90		
	High school	20	6.60	2.76		
	Vocational school	38	6.42	2.26		
	University/college	16	6.38	3.30		
	Total	232	5.47	2.90		
Risk Factors Knowledge	Below 8 grades	44	38.23	5.27	2.023	0.092
	8 grades	114	36.14	7.87		
	High school	20	37.90	6.87		
	Vocational school	38	35.95	9.46		
	University/college	16	32.50	3.97		
	Total	232	36.41	7.53		
Prevention Knowledge	Below 8 grades	44	4.50	1.80	2.691	0.032
	8 grades	114	3.67	2.18		
	High school	20	4.10	1.80		
	Vocational school	38	3.63	2.16		
	University/college	16	2.75	1.13		
	Total	232	3.79	2.05		
Barriers to seeking help	Below 8 grades	44	29.23	7.15	2.064	0.086
	8 grades	114	28.14	9.61		
	High school	20	29.70	9.50		
	Vocational school	38	27.00	11.62		
	University/college	16	22.13	4.24		
	Total	232	27.88	9.38		

*N*, sample size; SD, standard deviation; *F*, variability between the groups.

**Table 9:** Cancer Awareness Subscale Scores by employment status (one-way ANOVA).

Outcome	Employment	*N*	Mean	SD	*F*	*p*
Warning Signs Knowledge	Full-time job	44	7.32	1.84	7.944	0.000
	Prefer not to say	82	5.59	2.69		
	No job	17	4.76	2.63		
	Retired	8	6.75	1.39		
	Part-time job	57	4.05	3.09		
	Disability benefit	24	5.17	3.33		
	Total	232	5.47	2.90		
Risk Factors Knowledge	Full-time job	44	33.45	8.06	11.331	0.000
	Prefer not to say	82	37.80	6.30		
	No job	17	39.41	7.54		
	Retired	8	42.25	4.62		
	Part-time job	57	32.53	6.76		
	Disability benefit	24	42.17	6.08		
	Total	232	36.41	7.53		
Prevention Knowledge	Full-time job	44	3.55	1.87	4.265	0.001
	Prefer not to say	82	4.32	1.89		
	No job	17	2.88	2.03		
	Retired	8	2.50	2.20		
	Part-time job	57	3.32	2.09		
	Disability benefit	24	4.67	2.14		
	Total	232	3.79	2.05		
Barriers to seeking help	Full-time job	44	23.55	9.14	14.399	0.000
	Prefer not to say	82	30.17	7.77		
	No job	17	31.53	10.76		
	Retired	8	36.25	7.65		
	Part-time job	57	22.60	7.25		
	Disability benefit	24	35.17	8.66		
	Total	232	27.88	9.38		

*N*, sample size; SD, standard deviation; *F*, variability between the groups.

## 4 Discussion

This study aimed to adapt and validate a CAM tool for individuals with intellectual disabilities (ID) in Hungary and to assess their cancer literacy levels. The findings demonstrated that the adapted CAM possesses strong psychometric properties, confirming its reliability and validity for use in this population. Furthermore, this study provides valuable insights into the demographic and contextual factors influencing cancer knowledge and help-seeking behavior among individuals with ID. The adapted CAM tool showed satisfactory internal consistency across all subscales, with Cronbach’s *α* ranging from 0.714 to 0.842. These results align with previous validations of the CAM in the general population (1, 49), indicating that the core structure of the measure remains robust, even when it is adapted for individuals with ID. The Warning Signs and Barriers subscales exhibited particularly strong reliability (*α* = 0.842), suggesting that the items consistently captured the key aspects of symptom recognition and psychosocial barriers relevant to cancer awareness. Content validity analysis further confirmed the adequacy of the adapted instrument. The I-CVI values ranged from 0.79 to 1.00, with an average S-CVI/Ave of 0.96, surpassing the recommended threshold of 0.80 (48). This reflects a high level of expert agreement on the relevance and clarity of the items, particularly after linguistic and conceptual modifications to enhance accessibility for individuals with ID. The strong face and content validity results indicate that the tool is not only psychometrically sound but also comprehensible and appropriate for this population. Overall, this study identified modest levels of cancer literacy among the participants. Although awareness of warning signs was adequate, knowledge of risk factors and preventive behaviors remained limited. This finding is consistent with previous research, indicating that individuals with intellectual disabilities often have a restricted understanding of cancer-related information due to cognitive barriers and insufficient health education (8, 14, 50–54). The relatively higher internal consistency of the Prevention Knowledge subscale (*α* = 0.714) suggests that participants could reliably differentiate between healthy and unhealthy behavior. However, the mean scores indicated a significant potential for improvement in understanding cancer prevention concepts.

Association analysis revealed significant demographic differences between the groups. Individuals residing with family members exhibited a greater understanding of cancer warning signs than those in residential care. This pattern suggests that informal family support may enhance exposure to health-related discussions and community resources, whereas institutional environments may offer limited opportunities for personalized health communication (55–57). However, residents of care homes demonstrated slightly higher prevention knowledge, potentially reflecting structured health promotion programs or organized screening efforts within institutional settings (56). Interestingly, although residents of care homes demonstrated relatively higher prevention knowledge, they also reported higher scores on the barriers to seeking help subscale. It is important to note that higher scores on this subscale indicate greater perceived barriers to seeking medical care, rather than improved health literacy. This seemingly paradoxical pattern may reflect the complex influence of institutional living environments. Individuals residing in care homes may receive more structured health education or guidance from caregivers and organized health promotion activities, which could enhance their awareness of preventive behaviors. However, institutional contexts may simultaneously introduce structural barriers to accessing healthcare services, such as reliance on caregivers to arrange appointments, transportation limitations, or administrative procedures within care facilities. These findings suggest that improving cancer knowledge alone may not be sufficient to reduce barriers to care and highlight the importance of addressing structural and organizational factors when designing interventions to promote early help-seeking among individuals with intellectual disabilities. Participants with a family history of cancer showed significantly higher awareness of warning signs and prevention strategies and reported fewer barriers to seeking help. This finding aligns with previous research suggesting that personal or familial exposure to cancer increases perceived susceptibility and engagement in preventive behavior (58). Similarly, individuals who had previously participated in cancer screening exhibited better recognition of early symptoms, underscoring the educational role of screening programs in enhancing cancer literacy. Age, education, and employment status were important determinants of awareness. Older adults (≥65 years) displayed greater knowledge of cancer warning signs, possibly because of increased contact with healthcare services and exposure to health information over time. Higher educational attainment, particularly vocational or higher education, was associated with greater cancer knowledge, consistent with the well-established link between health literacy and education level (13, 57). Furthermore, full-time employees scored the highest in all knowledge domains, suggesting that engagement in structured work environments may enhance access to information and health-promoting communications. Notably, no significant sex differences were observed across the CAM subscales, in contrast to studies in the general population, where women typically reported higher cancer awareness (49, 59). This finding may reflect more homogeneous access to information and limited gendered health messaging within the ID population, highlighting the need for tailored, inclusive education strategies that consider cognitive and communication barriers rather than gender alone (60, 61). Moreover, training healthcare providers is essential for improving cancer awareness and promoting early detection in individuals with intellectual disabilities (ID). However, many caregivers and healthcare providers lack adequate training in cancer prevention and health promotion for this population (62, 63).

### 4.1 Implications for practice and future research

The findings highlight the adapted CAM as a reliable and valid tool for assessing cancer awareness among individuals with intellectual disabilities, representing a significant advancement in its accessibility. This instrument can lay the groundwork for targeted health education interventions aimed at improving cancer literacy and encouraging timely help-seeking behaviors. Given the observed disparities based on living arrangements and educational levels, future interventions should focus on inclusive communication, caregiver involvement, and the use of easy-to-read materials and visual aids. Additionally, the link between screening participation and awareness underscores the potential for integrating cancer education into preventive services. Health professionals and caregivers are crucial in reinforcing key messages and overcoming perceived barriers to seeking help (52, 64, 65). Further research should explore the predictive validity of the adapted CAM by examining its association with actual health behaviors and screening uptake. Longitudinal studies could also assess whether tailored interventions based on CAM findings lead to measurable improvements in cancer literacy and early detection in individuals with intellectual disabilities.

### 4.2 Strengths and limitations

This study is among the first to adapt and validate the Cancer Awareness Measure (CAM) for individuals with intellectual disabilities globally, thereby addressing an important methodological gap in assessing cancer literacy in this population. Including both community-dwelling and institutionalized participants enhanced the applicability of the findings across different living environments. However, several limitations should be acknowledged. First, the cross-sectional design precludes causal interpretation of the observed associations. Second, participants were recruited using a convenience sampling strategy through organizations affiliated with the Hungarian National Association of People with Intellectual Disabilities (ÉFOÉSZ). Although this approach facilitated access to the target population for tool validation, it may limit the representativeness of the sample and may over-represent individuals who are already connected to support services and potentially more exposed to health-related information. Additionally, the study included only individuals with mild to moderate intellectual disabilities, as participation required the ability to complete structured interviews. Consequently, the findings may not be generalizable to individuals with severe or profound intellectual disabilities, who may face different barriers to health information and healthcare access. The sample was also geographically restricted to Hungary, which may limit the transferability of some findings (e.g., screening behaviors) to other healthcare contexts. Furthermore, the use of self-reported data may be subject to comprehension variability or social desirability bias despite the adapted administration procedures. Finally, while the adapted CAM demonstrated strong content validity and internal consistency, additional psychometric evaluations, such as test–retest reliability and construct validity using factor analysis, should be conducted in future studies to further strengthen the measurement properties of the instrument.

## 5 Conclusion

In conclusion, the adapted CAM demonstrated strong reliability and outstanding content validity, confirming its suitability for assessing cancer awareness among individuals with intellectual disabilities in Hungary. The findings revealed significant disparities in cancer knowledge linked to demographic and contextual factors, underscoring the need for accessible, inclusive, and tailored health education intervention. This validated instrument lays the groundwork for future research and public health initiatives aimed at improving cancer literacy and promoting equitable cancer prevention among individuals with intellectual disabilities.

## Data Availability

The raw data supporting the conclusions of this article will be made available by the authors, without undue reservation.
